# Resorcin as a Dressing

**Published:** 1883-11

**Authors:** 


### Resorcin as a Dressing.

This new remedy promises to become not only the popular remedy for a number of ailments, but also to take the field as a dressing for chancres, chancroids, mucous patches, etc. It is said to be more efficient than iodoform, while it is free from the unpleasant odor of that drug. It may be applied in powder, or in twenty-five per cent solution in water.—*Canada Lancet.*

